# Emergence of behavioral phenomena and adaptation effects in human numerosity decoder using recurrent neural networks

**DOI:** 10.1038/s41598-023-44535-3

**Published:** 2023-11-10

**Authors:** Bhavesh K Verma, Rakesh Sengupta

**Affiliations:** 1 Indian Institute of Science Education and Research, Pune, 411008 India; 2 Center for Creative Cognition. SR University, Warangal, 506371 India

**Keywords:** Computational models, Computational neuroscience, Image processing

## Abstract

Humans possess an innate ability to visually perceive numerosities, which refers to the cardinality of a set. Numerous studies indicate that the lateral intraparietal cortex (LIP) and other intraparietal sulcus (IPS) regions (region) of the brain contain the neurological substrates responsible for number processing. Existing computational models of number perception often focus on a limited range of numbers and fail to account for important behavioral characteristics like adaptation effects, despite simulating fundamental aspects such as size and distance effects. To address these limitations, our study develops (introduces) a novel computational model of number perception utilizing a network of neurons with self-excitatory and mutual inhibitory properties. Our approach assumes that the mean activation of the network at steady state can encode numerosity by exhibiting a monotonically increasing relationship with the input variable set size. By optimizing the total number of inhibition strengths required, we achieve coverage of the full range of numbers through three distinct intervals: 1 to 4, 5 to 17, and 21 to 50. Remarkably, this division aligns closely with the breakpoints in numerosity perception identified in behavioral studies. Furthermore, our study develops a method for decoding the mean activation into a continuous scale of numbers spanning from 1 to 50. Additionally, we propose a mechanism for dynamically selecting the inhibition strength based on current inputs, enabling the network to operate effectively across an extended (entire) range of numerosities. Our model not only sheds new light on the generation of diverse behavioral phenomena in the brain but also elucidates how continuous visual attributes and adaptation effects influence perceived numerosity.

## Introduction

While our comprehension of numbers and mathematics relies on acquired knowledge and semantics, the remarkable ability to perceive small numerosities is observed even in infants and animals^1–3^. These findings provide compelling evidence for the existence of an innate mechanism for number perception within the human brain. Furthermore, studies utilizing lesion and brain imaging techniques have consistently identified a specific neural substrate situated in the left and right intraparietal areas, which is closely associated with numerical knowledge and relational understanding^4^.

Various behavioral observations, such as the accuracy, confidence, and response time of visual enumeration, have led to the classification of numbers into distinct ranges. The range spanning from 1 to 4 (sometimes 3 or 5) is commonly known as the “subitizing range”^5^. Within this range, humans exhibit exceptional speed and accuracy in rapidly enumerating a set of objects. On the other hand, larger numbers, typically from five onward, fall into the “estimation range.” In this range, individuals have two approaches to enumeration: counting, which is a slower but highly accurate process, or estimation, which is a much quicker process but it comes with some level of error^5,6^. The magnitude of error in estimation increases proportionally with the number of items to be estimated, consistent with Weber’s law^7,8^.

The processes involved in enumerating numbers within the subitizing range and estimation range have sparked considerable debate. The enduring nature of subitizing despite disruptions has led to the notion that it may be preattentive, or at the very least rely on preattentive information^9^. However, some studies have presented evidence that subitizing is indeed susceptible to attentional load^10–12^. Furthermore, supporting the existence of distinct mechanisms for large and small numbers, some studies have proposed a connection between subitization and object individuation: a visuospatial mechanism enabling us to locate and track a limited set of objects^13,14^, while others have suggested a pattern recognition model for subitization^15,16^.

The existence of two distinct processes for subitizing and estimation has been brought into question by several behavioral studies. Weber fractions for the perception of numerosity in the estimation range are reported to be around 0.25^17^. Due to the error-less nature of enumeration within the subitizing range, the calculation of the Weber fraction is not feasible. However, if we make the assumption that the subitizing range shares the same Weber fraction of 0.25 as the larger numbers, we can calculate the ’just noticeable difference’ for numbers within the subitizing range. This calculation yields a value less than or equals to one (4 multiplied by 0.25 equals 1). We observe that the just noticeable difference within the subitizing range is smaller than the difference between two closest stimuli (numbers have the least count of one). This indicates that distinguishing between any two numbers in the subitizing range is remarkably effortless. Consequently, an argument arises that the error-free nature of enumeration within the subitizing range could be attributed to the enhanced resolution, implying that the underlying mechanism might be the same for perception of both small and large numbers^18,19^. Furthermore, Balakrishnan and Ashby^20^ did not find a sharp discontinuity in reaction times between the estimation and subtitling ranges. Moreover, a recent study by Portley and Durgin^21^ introduces a new inflection point in the estimation range, further dividing the larger numbers into two distinct ranges: below and above 20. This finding further complicates the classification of numbers^21^.

Nieder et al.^22^ demonstrated the presence of ’number neutrons’ in the Lateral Prefrontal Cortex (IPFC) and Intraparietal Sulcus (IPS) through their experiments that involved training monkeys to discriminate numerosity using the Delayed Match-to-Sample (DMS) paradigm. These number neurons exhibited a clear tuning function for numerosity, with the highest firing rate occurring for the preferred numerosity and decreasing as the numerical distance increased^22,23^ . Another study by Roitman et al.^24^ examined the activity of individual LIP neurons in monkeys while viewing dots arrays on a computer screen. They found that these neurons responded proportionately to the number of elements in the display over an extended range of numerosities. The observed monotonic increase in neuronal activity with numerosity supports the existence of an integration stage. In our model as well, we employ a recurrent neural network which encodes numerosity as the monotonically increasing mean activation of the network.

Dehaene and Changeux^18^ has provided us with a foundational framework to conceptualize different stages of number processing within Approximate Number System (ANS) paradigm. The first stage is the sensory stage, where the sensory properties of stimuli, such as size, color, density, and convex hull, are processed. To address potential confounding factors, the sensory information is then transferred to the normalization stage. In this stage, the confounding sensory information is removed, and only pure numerical information is retained. The numerical information is then passed on to the accumulator stage. Researchers frequently use the analogy of a beaker pouring water into a cylinder to explain this stage. Where the water in the beaker represents the normalized sensory input for individual objects and the water in the cylinder represents the total magnitude of the total numerosity. However, because the beaker does not always contain the same amount of water, the total amount of water in the cylinder grows more inaccurate with each pour. As a result, the final estimate of numerosity at this stage is ’approximate’. The decline in accuracy of the estimated numerosity with an increasing number of pours aligns with the observation that performance in discriminating numerosity decreases as the numerosities increase, indicating a ratio-dependent performance.

In contrast to ANS the Sensory Integration Theory (SIT) argues that the brain derives numerosity from other visual cues such as size and density^25,26^. Dakin et al.^26^ employ spatial filtering techniques to generate summary statistics of a stimulus, with the aim of simulating both numerosity and texture discrimination. Their approach involves normalizing the high spatial frequency content with the low frequency content in the stimulus. The estimation of these contents is obtained by applying filtering techniques using small and larger Laplacian of Gaussian filters, respectively. By selecting an appropriate filter size, the high frequency response demonstrates a proportional increase with set size, which corresponds to the number of elements. In contrast, the low-frequency estimator exhibits hybrid behavior, growing moderately with set size and area. This implementation embodies a fundamental principle: with a fixed element size, the number of items is correlated with the amount of borders. Therefore, number judgments can be made simply by examining the high-frequency content. Although the authors do not specifically address sensitivity, given that the primary limitation of the model is stimulus-based noise, one would expect a square-root relationship rather than adherence to Weber’s law.

In recent years, several models have been introduced for number perception, employing backpropagation-based artificial neural networks. Because of its effectiveness in tasks such as classification, decision-making, and learning, artificial neural networks has grown in popularity^27,28^. These networks learn by adjusting the weights between neurons to minimize errors, as determined by a cost function. However, despite their utility, backpropagation-based artificial neural networks often face criticism from the scientific community for their limited ability to provide insight into biological mechanisms. To advance our understanding of number perception, it is crucial to develop computational models that are biologically relevant and capable of simulating key behavioral findings related to visual number perception.

In the initial part of this study, we propose a possible mechanism for achieving normalization stage of the ANS theory to obtain an output comparable to an Object Location Map (OLM)^18^. Rather than utilizing visual information in its complete complexity, we propose harnessing the power of a saliency map to extract numerosity. By employing a saliency map, we can effectively extract the relevant information necessary for quantifying numerical magnitudes, avoiding the need to process the visual input in its entirety. Saliency maps represent potentially important locations in the visual field and are usually represented as a heat map. It’s a common consensus that the ventral stream mainly subserves the recognition and discrimination of objects and works on smaller visual fields. While the dorsal stream is associated with the detection of location and eye movements, it covers a larger visual field^29^. Visual enumeration often works on a wider visual angle. In this context, the role of the dorsal stream becomes crucial, as enumeration tasks often necessitate the accumulation of information from various regions of interest. We begin with the assumption that, in scenarios where objects in the visual field are spatially separated, information from a saliency map alone may suffice to extract numerosity. To illustrate the concept, we generate normalized information akin to OLM (Object Location Map) using saliency map of an image.

As a model of the integration stage, we use an RNN previously studied in Sengupta et al.^30^. We use the normalized input (similar to OLM) scheme for the RNN. The output of the network is determined by calculating the mean activation of its nodes at steady-state. We demonstrate that a single neural network has the ability to adapt its internal parameters, specifically the inhibition strength, effectively handling both small and large numbers. This notion is supported by the number perception model proposed by Sengupta et al.^30^. Inspired by the Normalized Object Location Map (OLM) employed in Dehaene and Changeux^18^, we incorporate a similar input scheme for the RNN.

Our model successfully replicates key findings from behavioral studies, including the Weber fraction, number comparison, and reaction time. Building on the model proposed by Sengupta et al.^30^, we introduce a method to decode the network output (mean activation) into numerical estimations. To ensure the network’s applicability across the extended range of numbers (1 to 50), we employ multiple inhibition strengths. By optimizing the total number of inhibition strengths required, we identify three specific inhibition strengths (0.01, 0.04, and 0.15) that correspond to three distinct ranges of numbers (1:4, 5:17, and 21:50). The emergence of different numbers ranges as a result of optimizing a single network offers a fresh perspective on interpreting the mechanisms underlying the observed inflection points in numerosity perception. We conclude our study by suggesting possible mechanisms by which network inhibitions can be regulated based on the current input and how the adaptation effects relate to it.

## Methods

### Using saliency map for the normalization stage

**Figure 1:**
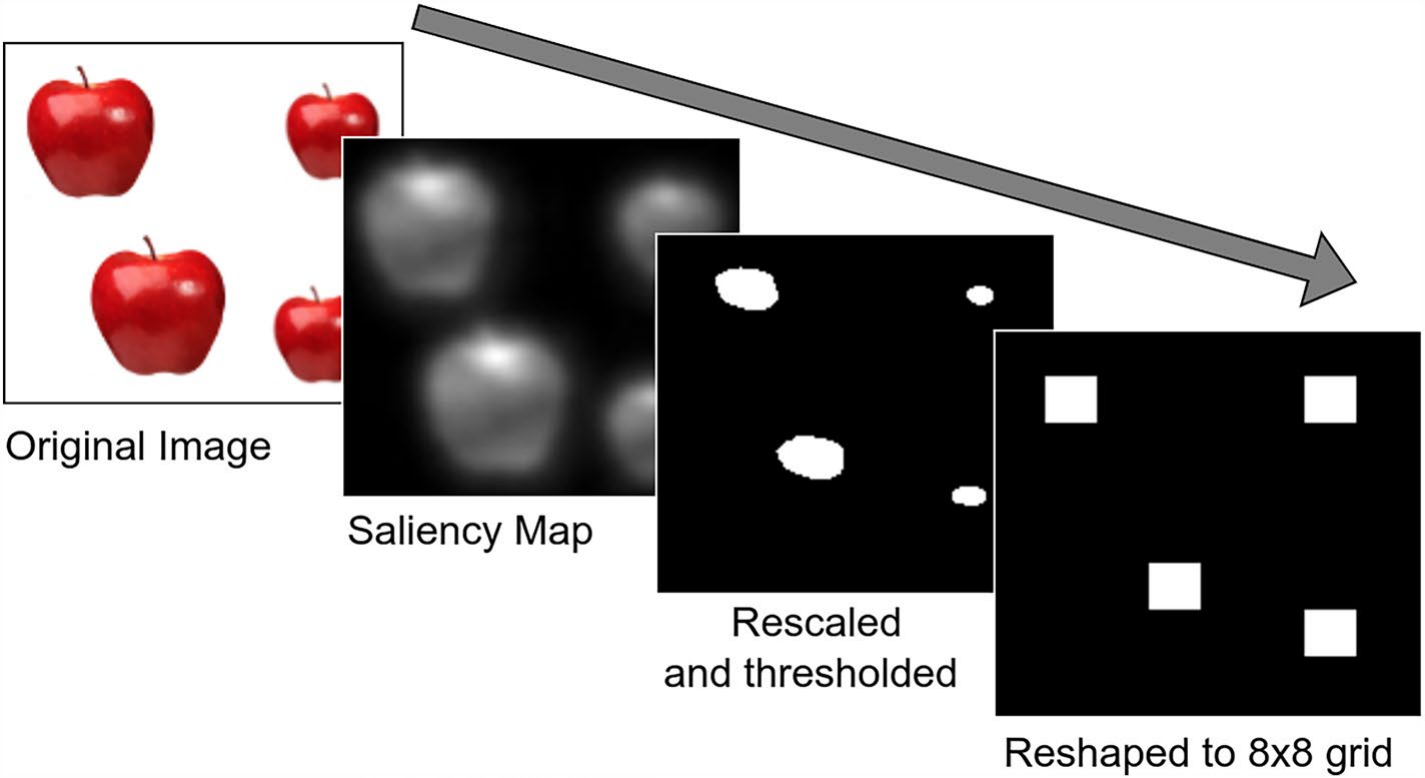
Extraction of Object Location Map (OLM) from an image using saliency map This diagram depicts the utility of saliency map in the process of normalization of visual information to obtain a numerosity sensitive data similar to Object Location Map. We can see that the use of saliency maps drastically simplify the process by extracting spacial distribution of the objects in a visual scene. The objects are represented as a set of patches that lose their size and shape dependency in each step of the normalization and finally result in a data similar to OLM.

In this section, we demonstrate that the use of a saliency map can facilitate the normalization stage to obtain numerosity sensitive information, similar to Object Location Maps (OLM). First, we take an image with spatially separated objects and generate its saliency map using Rare2012^31^ saliency model (Fig. 1). After generating the saliency map, we discard all visual information other than the saliency map for further processing. By using the saliency map, we gain a significant advantage in simplifying the subsequent normalization stage. When using an image with its full complexity, the normalization stage presents a challenging task of filtering out various confounding visual attributes from the sensory stage, including color, size, convex hull, intensity. However, with the incorporation of the saliency map, this task becomes more straightforward. We expect the saliency map to capture the objects in a visual scene in the form of bright patches. Hence, the normalization stage now primarily involves removing size and shape information from the patches. Ideally, a perfect normalization process would yield a collection of binary data streams, with each active unit representing a single patch on the saliency map. In this situation, the process of normalization might just involve the tasks of finding contiguity and local maxima in the saliency map. Here, we have utilized some simple operations using OpenCV to visually demonstrate the concept (Fig. 1). First, we rescaled the data to values from 0 to 1 and then applied a threshold with a value of 30 as the parameter by using CV.threshold method, which removed salient regions highlighting the peak intensities. We obtained four patches corresponding to the four objects present in the image. At the last stage, we have reshaped the data into an 8Œ8 grid to match the dimension of the input to our RNN, which has 64 neurons arranged in an 8Œ8 grid.

### Network architecture

**Figure 2:**
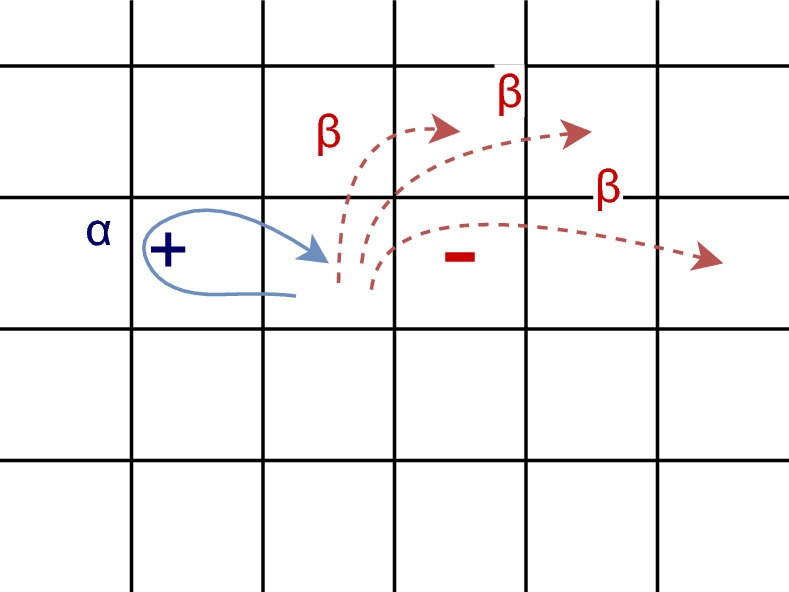
Network Diagram Squares in the diagram represent neurons in the network. Our network consists of a single layer of 64 neurons. Each neuron has an excitatory connection (blue arrow) with itself and inhibitory connections (red arrows) with all other neurons in the network, where $\alpha$ denotes excitation strength and $\beta$ denotes Inhibition strength.

Our model consists of an on-center off-surround recurrent network of 64 neurons (Fig. 2), where every neuron has an excitatory connection with itself and inhibitory connections with all other neurons. We are using the same network extensively studied in Sengupta et al.^30^. The network is symmetric in nature and is parameterized by three variables: self-excitation strength ($\alpha$), mutual inhibition strength ($\beta$), and decay constant ($\lambda$) (Table 1). The dynamics of the network is governed by Eq. (1).

**Table 1:** Network dynamics simulation parameters.

Parameter	Value
N (number of neurons)	64
$\alpha$ (strength of self excitation)	2.2
$\beta$ (strength of mutual inhibition)	0.01–0.15
Duration of stimulus presentation (in time steps)	100
Total duration of simulation (in time steps)	5000

1 $$\begin{aligned} \frac{dx_{i}}{dt} = -\lambda x_{i} +\alpha F( x_{i}) -\beta \ \sum _{j=1,j\ne i}^{N} F( x_{j}) \ +I_{i} +\ Noise \end{aligned}$$

$x_{i}(t)$ (written as $x_{i}$) stands for activation of the i-th node at time t, $I_{i}(t)$ (in short $I_{i}$) is externally injected input current, which has value 1 when the i-th node is presented with an input for an initial presentation time, and zero for the rest of the simulation. The activation function follows the formula:

2 $$\begin{aligned} F(x) ={\left\{ \begin{array}{ll} \ \ \ 0 &{} for\ x\leqslant 0\\ \frac{x}{1+x} &{} for\ x >0 \end{array}\right. } \end{aligned}$$

The noise is sampled from a normal distribution with a mean of 0 and a standard deviation of 0.03. Since changing the excitation parameter does not affect the general nature of the data^30^, the self-excitation strength is fixed at the value of 2.2. We have kept an inventory of inhibition strengths to pick from, ranging from 0.01 to 0.15. The number of neurons receiving an input current during the initial presentation time is referred to as the “set size”. The set size represents the numerosity of a stimulus. To simulate the neural dynamics, we have used Euler’s method in Matlab software. During the presentation time, which is kept much smaller than the overall duration of the simulation (time steps = 100 compared to 5000 for the full simulation), the nodes assigned to the set size are injected with an input current (Ii) valued at 1.

When we simulate the network dynamics of the RNN network using Eq. (1), we obtain a pattern of activation across its 64 neurons as the network reaches a steady state. This steady-state activation pattern of the network nodes depends both on the set size (as the input corresponding to a numerosity) and the inhibition strength (as a network parameter). Using Eq. (3), we calculate the network’s mean activation (*MA*) from the steady state activation and use it as the main output from the RNN.

3 $$\begin{aligned} \displaystyle MA =\frac{1}{N}\sum _{i=1}^{N} x_{i} \ \end{aligned}$$

**Figure 3:**
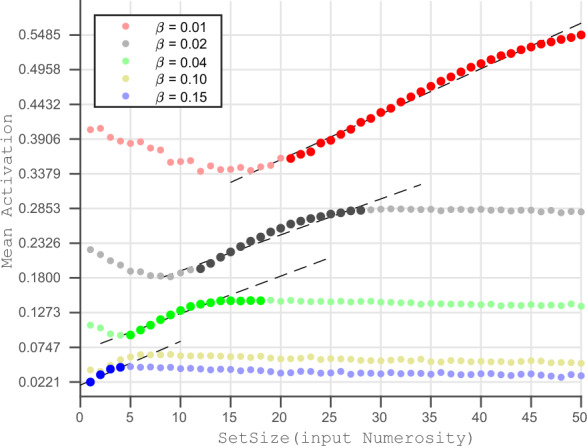
Input Numerosity Vs Mean Activation of Network, A plot between set size (Input Numerosity) and corresponding Mean Activation for five of the ten inhibition strengths. The darker spots denote the data points where Mean Activation increases monotonically with set size (input numerosity). The black dashed lines are the linear fit between the two variables, taking data only from the monotonic regions. We can observe that for strong inhibition ($\beta =0.15$, blue) mean activation increases monotonically with small numerosities while for weak inhibition ($\beta =0.01$, red) mean activation increases monotonically with larger numerosities. The curves for $\beta =0.10$ and $\beta =0.15$ and all other intermediate inhibition strengths (not plotted here) follow a qualitatively similar pattern. $\beta =0.10$ $\beta =0.15$. We generated the data for this plot by replicating the simulations done in Sengupta et al.^30^ with an improved resolution.

After taking the average of mean activation from 30 simulations for each combination of set size and inhibition strength, we plot the mean activation against set size for different inhibition strengths (Fig. 3).

### Decoding mean activation into number estimates

Sengupta et al.^30^ successfully demonstrate that the mean activation encodes information about the numerosity. By directly comparing the mean activation generated by various inputs, they simulate behaviorally significant outcomes such as the size and distance effect. The study shows that, with this technique, the magnitude of the inputs can be compared without converting the corresponding mean activation into numerical estimates. In this section, we propose a method to decode mean activation to corresponding numerical estimates, which not only allows us to compare the magnitudes of two numerosities, but also obtain number estimates for individual inputs. First, we run network dynamics simulations using all combinations of set size (1-50) and 15 inhibition strengths (0.01-0.15) 30 times and calculate an average of the mean activation resulting (as in Fig. 3). For each inhibition strength, we take data points mapping the set size to mean activation solely from monotonic regions and fit a line between them. four of the fifteen linear fits are shown in Fig. 3 as dotted lines. This gives us 15 functional relations ($\displaystyle LinearFunctions_{\beta }$) corresponding to each of the 15 inhibition strengths ($\beta$) used for simulations. By taking the inverse of these linear equations, we obtain linear equations ($\displaystyle F_{\beta }^{-1}$) mapping each mean activation to number estimates depending on the inhibition strength used for simulation. To obtain an estimation of numerosity from a given mean activation, we use the inverse of these linear functions ($\displaystyle F_{\beta }^{-1}$) to map mean activation to corresponding number estimation.

$$\begin{aligned}Number\ Estimate &= F_{\beta }^{-1}(MA)\\\quad Estimation\ Error&=Number\ Estimate-set\ size \end{aligned}$$

It’s important to note that the output from the network (mean activation) is not just a value created by a formula applied on an input (set size). Instead, the mean activation is a statistic (mean of all neuronal activations) calculated from the steady state activity of nonlinear dynamics. So it is not possible to directly find an inverse function that maps the mean activation to the corresponding set size. The functions that we are using to decode the mean activation are inverse functions ($\displaystyle F_{\beta }^{-1}$) of the learned relation between numerosity and mean activation obtained from linear fits between the two variables using simulated data points only from monotonic regions. Each of these linear functions is indexed by the inhibition strength that was used to generate the training data for the linear fitting. Therefore, the decoding function ($\displaystyle F_{\beta }^{-1}$) operates exclusively with the newly acquired mean activation value generated using the identical inhibition strength. For example, if we acquire a mean activation of 0.53 using a set size of 5 as input for a network with an inhibition strength of 0.15, to obtain the numerical estimate of the mean activation, we use the inverse function already obtained corresponding to the inhibition strength ($\beta$) of 0.15.

$$\begin{aligned}Number\ Estimate&=F_{0.15}^{-1}(0.53)=4.82\\\quad Estimation\ Error&=4.82-5=-0.18 \end{aligned}$$

**Figure 4:**
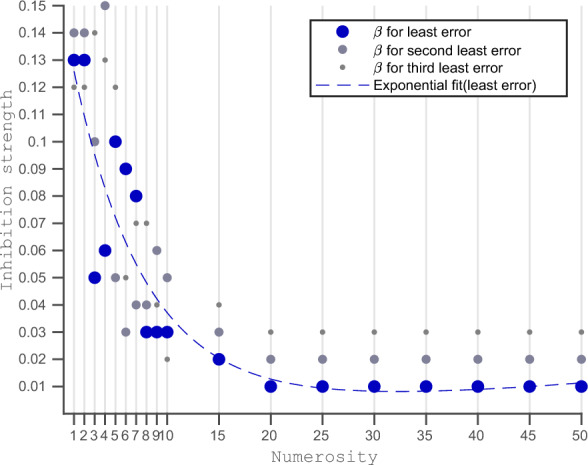
Numerosity Vs Inhibition Strengths Producing Lowest Error Using fifteen network inhibition levels, we obtain fifteen different number estimates for an input. For each input numerosity (x-axis), we plot the three inhibition strengths that result in the three lowest errors (y-axis). We fitted an exponentially decreasing curve to show the pattern between input numerosity and the mean activations leading to the least amount of error. For smaller numbers, high inhibition strength results in a minor error in estimation, and vice versa. We can see that for input numerosity 1, an inhibition strength of 0.13 results in the lowest error, while for input numerosity 50, an inhibition strength of 0.01 results in the lowest error.

**Figure 5:**
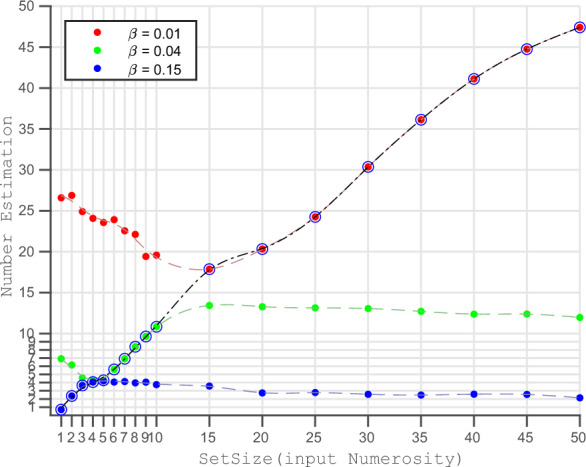
Input Numerosity Vs Number Estimations For three inhibition strengths, set size (input numerosity) is plotted against the corresponding number estimates. Network with high inhibition (0.15) estimates small numbers (1–4) accurately but underestimates larger numbers. None of the inhibition strengths are effective across the full range of numerosities. We obtain a curve (black) that can provide reasonable estimation for the extended range of numerosity (1–50) by varying the inhibition strength depending on the input (Selection Algorithm).

Similarly, we obtain ten different number estimates for the same input set size of 5 by employing ten different inhibition strengths. To demonstrate the results of this method, we plotted number estimations against set size for three different inhibition strengths (0.01, 0.04, and 0.15) in Fig. 5). To understand the general pattern of inhibition strength leading to the lowest amount of errors, we plotted inhibition strengths leading to three of the smallest estimation errors against input numerosity (Figure 4).

## Result

### Emergence of different ranges of numbers

By simulating the network dynamics using fifteen different levels of inhibition using the inputs from set size 1 to 50, we obtain data-points mapping set size to mean activation corresponding to each of the inhibition strengths. We took the average of 30 simulations for each data point and plotted the relationship between the set size and the mean activation for five inhibition strengths (Fig. 3). In the figure, we can see that the input numerosity and mean activation follow different relationships depending on the inhibition strength used for simulation. For a very strong inhibition ($\beta =0.10$ to $\beta = 0.15$), the relationship is quite similar, where mean activation increases with set size until numbers 4 to 7, then slowly decreases with an almost flat curve for larger numerosities. Whereas for medium to low inhibition strengths ($\beta =0.01$, $\beta =0.02$, $\beta =0.04$ in the plot), first mean activation decreases with an increase in set size, then it increases monotonically for a range of numerosities (denoted by darker dots in the plot) followed by a flatter region for even larger numerosities. We observe that the monotonic regions cover larger numbers in cases of smaller inhibition strengths and vice versa. By minimizing the number of different inhibition strengths required so that their combined monotonic regions cover the full stretch of numerosity, we ended up with three inhibition strengths ($\beta =0.01$, $\beta =0.04$, $\beta =0.15$) and three ranges of numbers (1:4, 5:17, and 21:50) corresponding to them.

Behavioral studies demonstrate the presence of separate intervals of numbers based on differences in their psychophysical properties (or numerical cognition effects), such as accuracy and reaction time^5,6^. The differences between the subitizing range (from 1 to 4) and the estimation range (greater than 5) are among the most studied observations in number perception research. In a recent study, Portley and Durgin^21^ suggested the presence of another inflection point in the number-estimation range, further dividing the estimation range into two intervals, below and above number 20. Our model simulates the emergence of these number intervals with surprisingly similar boundaries as a result of the minimal coverage of the monotonic relation between network input (set size) and output (mean activation).

### Estimation of numerosity

When we examine the error patterns for all combinations of set size and inhibition strength (Fig. 5), we discover that no inhibition strength produces accurate estimation across all the available numbers. We observe that using higher inhibition strengths result in fewer errors in the case of small numbers, while lower inhibitions work best for larger numbers. For example, as seen in Fig. 5, a high inhibition (0.15) network accurately estimates small numbers (1–5) and underestimates larger numbers (6–50). The relatively lower inhibition (0.04) network overestimates the small numbers (1–5) and produces accurate estimates for larger numbers (6–20).

In Fig. 4, we observe that for a larger numerosity, the inhibition strength leading to the least amount of error (large blue dots) is consistently equal to 0.01. While the inhibition strengths corresponding to the second and third-lowest errors are progressively stronger ones (0.02 and 0.03). For the smaller numbers in the range of one to ten, the inhibition strengths responsible for the lowest errors are not very consistent. However, we see that for a smaller number, on average, a larger inhibition strength produces smaller errors.

In psychophysics research, it is a general practice to examine whether a stimulus is perceived as greater (overestimation) or lesser (underestimation) than its actual magnitude. In Fig. 5, we see that while using a high inhibition strength (0.15), our model underestimates larger numerosities (greater than 5). Whereas using a low inhibition strength (0.01) it overestimates smaller numbers (1–20). When we ignore all the regions where input numerosity and number estimation have a negative correlation (negative slope), we see that for any inhibition strength, every numerosity either gets estimated correctly or underestimated. This result has some important implications for adaptation effects, which are analyzed in depth in a subsequent section.

### Regulation of network inhibition

In the previous section, we offered a technique for extracting numerosity information from the network’s mean activation. We demonstrated that, depending on the input’s numerosity, using some levels of network inhibition results in better number estimation than others. In this section, we will investigate a ’selection algorithm’ to control network inhibition, allowing the same network to generate the best possible estimates across the extended range of numbers.

Through Figs. 3 and 4, we can see the importance of monotonicity for accuracy of estimation. In Fig. 3, high inhibition strength (0.15) produces monotonicity for numerosities ranging from 1 to 4. Similarly, in Fig. 4, we see that high inhibition strengths (0.15, 0.14, and 0.13) result in the lowest errors for those numerosities. When we assign the network inhibition strength based on the interval in which the input numerosity falls, we obtain a favorable estimate. For example, if we have the current input with numerosity of 2, we can use the inhibition strengths of 0.13 to 0.15, as with these inhibition strengths, the output of the network increases monotonically with input. Using these inhibition strengths gives us an estimation with the most minor error, as we see in Fig. 4; for numerosity of 2, high inhibition strength produces the smallest errors. Although this illustration of the selection of inhibition strength aids our understanding of the model, there is a fundamental reason why it cannot serve as a stand-alone model for network regulation. Here, we have used a specific inhibition strength based on the input’s numerosity. However, because number estimation is the last step, the system does not know the current input’s numerosity when choosing the inhibition level. We have implemented a sensitivity-based algorithm to choose a suitable inhibition strength, rather than choosing the inhibition based on the information of numerosity.

To illustrate the concept, we only use a previously obtained set of three inhibition strengths, which guarantees that we have at least one inhibition strength where the condition of monotonicity is satisfied for every potential input to the network. For a given input set size, we determine the sensitivity of the output (number estimate) to the change in input. The sensitivity is obtained by measuring the slope of the curve between the two variables. We obtain three values for the sensitivities corresponding to three levels of inhibition strengths. The algorithm then selects the level of inhibition for which the sensitivity is the highest. For instance, for the input of set size 2, we can see in Fig. 3 that the blue curve corresponding to inhibition strength 0.15 has the highest slope (all other slopes have negative values in the region). As a result, the algorithm chooses the inhibition strength of 0.15 to be the most suitable for the input. We refer to this process as the ’Selection Algorithm’. we obtain the estimate of 1.9 using the network inhibition strength of 0.15, which is more precise than the estimates we would have otherwise obtained using the other inhibition strengths.

We plotted the set size against the number estimations using the three different inhibition strengths of 0.01, 0.04, and 0.15 (Fig. 5). Black circles in the figure show the data points obtained using the inhibition strengths chosen by the Selection Algorithm. As anticipated, we discover that for the extended range of inputs, the estimations using the chosen inhibition strength produce the most accurate number estimates compared to other inhibition strengths. Following the instance of input of set size 2, we obtain an estimation of 1.9 using the chosen inhibition (at blue curve in Fig. 5) whereas other inhibition strengths produce highly inaccurate estimations.

### Role of continuous attributes

**Figure 6:**
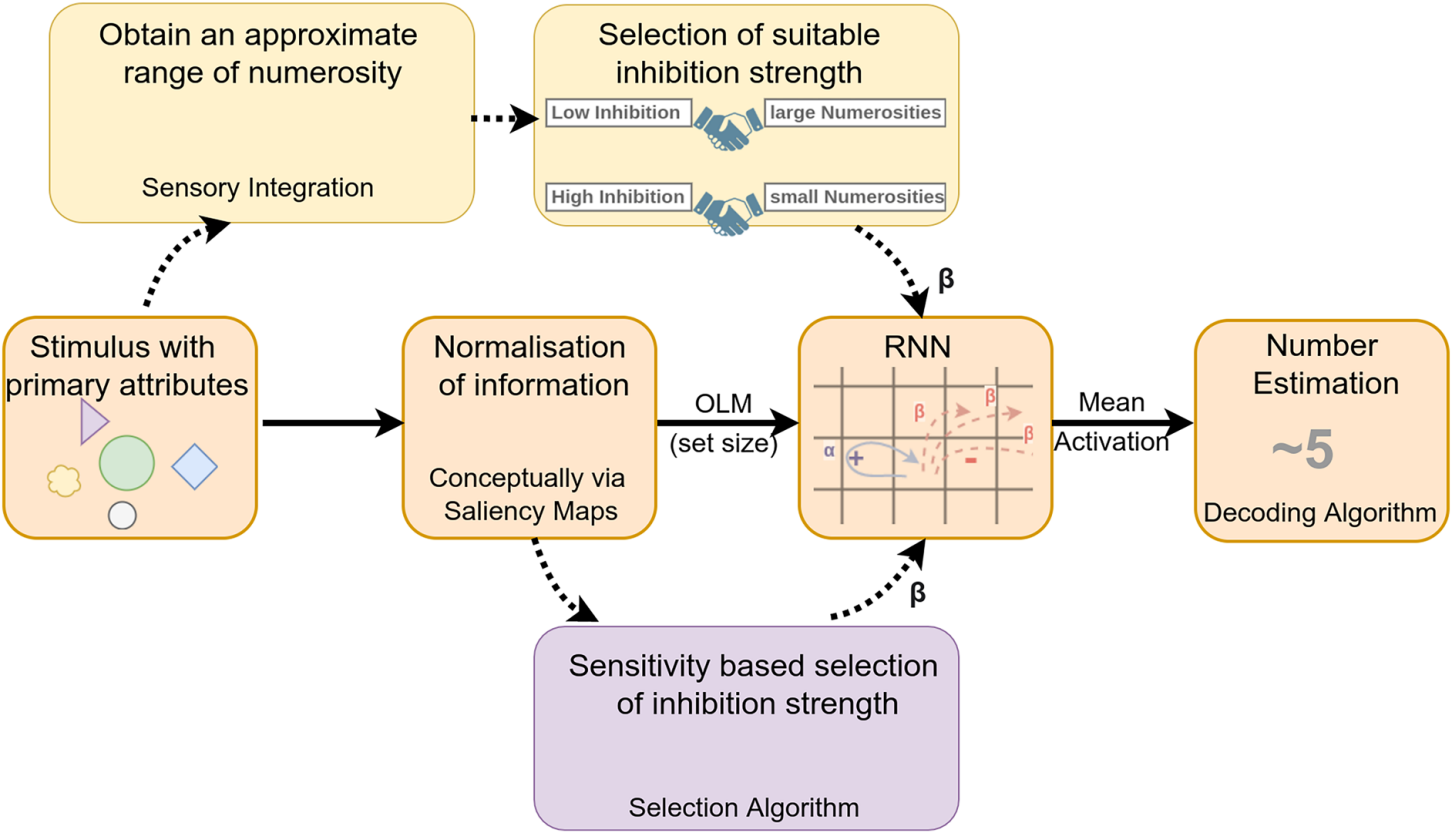
Model Overview This diagram depicts the flow of information in our model of visual perception at the central. First, let’s follow the central flow of information (solid arrows). We start with a stimulus with primary visual attributes. This crude information goes to the normalization stage, which extracts a normalized information similar to Object Location Map (OLM). In the next stage, we take the normalized information as the input to the RNN in the form of set size. The RNN gives the mean activation of its internal nodes as its main output. We have shown that the mean-activation of the network contains the information of numerosity. To decode the mean activation back to a magnitude (number estimate) that can be compared to its numerosity we use the Decoding Algorithm. One of our main findings is that the RNN needs to utilize different inhibition strengths to work for a wide range of numerosities. The yellow and blue boxes depict two possible ways to select the appropriate inhibition strength based on the current stimulus. Some studies have suggested that it is possible to extract approximate information on numerosity from the primary visual attributes^41^, the first yellow box denotes a possible mechanism to obtain an approximate range of numerosity. As using a lower inhibition gives the most accurate estimation for large numbers and vice versa, the system can theoretically decide the suitable inhibition strength by using this information of the approximate range of numerosity. On the other hand, the purple box denotes an alternate mechanism for choosing network inhibition. Using the Selection Algorithm, we show that choosing the inhibition strength that leads to the highest sensitivity of mean activation to a small modulation in input leads to a better internal representation of numerosity.

Number perception is influenced by other visual attributes of objects in a scene, such as density, size, and convex hull^32^. As discussed previously, the Sensory Integration Theory argues that the brain derives numerosity from various continuous cues^25,26^. Some studies have opposed the idea by arguing that sensory cues are insufficient in themselves to produce an accurate estimation of numerosity^33^. Even if the sensory cues are not enough to generate an accurate estimation of numerosity, they might still give a preliminary idea of how small or big the numerosity could be. We propose that information from sensory cues can be used to generate an initial guess about the possible number range of the input at a very early stage of the visual stream. This approximate information about the magnitude of numerosity can be used to set the context (here, by choosing an appropriate inhibition strength) for the integration stage, which finally leads to an accurate representation of numerosity. This idea is shown in Fig. 6, where the central flow of information (with solid arrows) depicts the information processing using our model within the ANS paradigm, and the flow of information in the upper section (with dashed arrows) illustrates the hypothetical process of selecting a suitable inhibition strength based on sensory integration of visual cues. Under this assumption, the widely recognized influences of the continuous attributes (such as size and density) on the perceived numerosity could be a result of two factors; first, an imperfection in the normalization of information leading to an incorrect Object Location Map (OLM), and second, an imperfection in the calculation of an initial guess by sensory integration leading to an error in the integration of the OLM.

### Adaptation

Psychophysics experiments have revealed that the perception of numerosity exhibits susceptibility to adaptation^34,35^. When humans are exposed to multiple stimulus with certain numerosity (“adaptor”) for an extended period, the apparent numerosity of a subsequent (“test”) stimulus gets distorted. Adaptation is commonly thought to be an hallmark of “primary” perceptual attributes of vision, such as color, orientation, size, or density. Similar to many of these primary perceptual attributes, perception of numerosity follows Weber’s law, which led many researchers to consider it as a “primary visual feature”^36^. In this study, we attempted to model high-level visual processing of numerosity that accepts input devoid of primary visual attributes. The variability of the network inhibition strength plays a vital role in our model because it allows for accurate internal representation of the whole range of numerosity. This section will discuss how the need for dynamic network parameters (here, inhibition strength) can relate to adaptation effects.

As discussed in previous sections, our model needs to employ different inhibition strengths for the network depending on the range of numerosity. Our model is very simplistic in nature and is far from the complexity of biological networks with multiple degrees of freedom. We can assume that regulating inhibition strengths or other network parameters can affect how accurately the brain represents numerosity internally. These processes might be reflected in behavioral observations because of the time and error associated with them. Assuming, with each instance of processing a certain range of numerosity, the network gets closer to the most appropriate inhibition strength; it would also increase the accuracy with each iteration. This way, we can draw a parallel between the adaptation of network parameters and the adaptation to numerosity.

Studies have shown that humans take longer (increase in reaction time) to enumerate or compare numerosity just after a switch from an adapted numerosity range. Additionally, the accuracy decreases immediately after the switch and then improves again with repeated exposure to the current range^37^. The additional time required to select an appropriate network parameter might have a connection to the “switch cost” in the form of a delay in response. Using a network parameter suitable for the previously adapted numerosity might have a relationship with the observed drop in accuracy immediately after the switch. Exploring the possibility of such adaptation mechanisms in the higher levels of the number processing pathway can help us go beyond the conceptualization of numerosity as a primary visual feature.

In Fig. 5 we see that the inhibition strength of 0.04 is suitable for numerosity between 6 and 20. Suppose the network is set to an inhibition of 0.04; let’s call it as ‘adapted’ to numbers in the range of 6 to 17. When we suddenly provide an input corresponding to a larger numerosity (say 30), ideally, the network should shift to a lower inhibition strength (0.01) to give an accurate result. But if it uses the same inhibition strength of 0.04, it would underestimate the numerosity to be around 20 (on the green curve). Similarly, whenever we use an inhibition strength adapted to smaller numerosity on inputs with larger numerosity, we obtain an error in the form of underestimation. But when we use inhibition strength adapted to larger numerosity for smaller numerosity, we obtain an overestimation of numerosity. The plot fails to demonstrate the underestimation observed in behavioral experiments while switching from larger to smaller numerosities^36,38^. But the regions in the plot which correspond to overestimation (numerosity from 1 to 10 on the red curve), the estimation of numerosity decreases with increase in input numerosity. As this does not follow the primary condition of monotonic increasing relationship between the input and the output, we discard this region.

Castaldi et al.^38^ classified the brain activity recorded during numerosity perception before and after psychophysical adaptation to numerosity using multivariate pattern recognition. Using a support vector machine, they used BOLD responses from the Intraparietal Sulcus (IPS) to classify numerosity successfully. Based on the observation that training the model with the pre-adaptation responses did not classify numerosity while testing on post-adaptation data and vice versa, they suggest that adaptation changes the neuronal representation of numerosity in the brain. Similarly, our model shows that the neuronal representation of numerosity changes with the inhibition strength. As shown in Fig. 3, we obtain different curves mapping the numerosities to the mean activations for each inhibition strength. As we have suggested that adaptation is essentially achieved by changing network inhibition, our model also supports the claim by Castaldi et al.^38^ that adaptation changes the neural representation of numerosity. They showed that the degree of adaptation increases the discriminability of the Intraparietal Sulcus (IPS) BOLD response. In our model, the mean activation of the network is used as the basis for the magnitude of numerosity. When using the most appropriate inhibition strength, the mean network activation is most sensitive (has the highest slope) to change in numerosity. We once again draw a comparison between adaptation and the control of network inhibition, and by observing that the greater slope of the mean activation leads to greater discriminability.

Previously, we discussed some possible ways to choose the inhibition of the network to suit individual inputs. We suggested that a method that can generate an approximate prediction of the range of numerosity can be used to choose inhibition strength for the network, which in turn gives a more accurate internal representation of numerosity. One of the ideas involved generating an approximation of numerosity by using continuous visual cues like size, density, and texture (The flow of information in the upper section of Fig. 6). In such a case, these continuous attributes indirectly control the internal representation of the numerosity. It is important to distinguish between this kind of influence and the sensory integration theory, which holds that the knowledge of numerosity is entirely derived from the sensory cues. Here, only the level of network inhibition is preconditioned by the sensory cues so that it is prepared to work with a normalized and segmented input. Durgin^39^ claimed that adaptation to texture density affects the perception of numerosity. They have argued that our brain does not adapt to numerosity but to the texture density of a stimulus. Other studies have also shown that adaptation to other visual cues like size also affect numerosity perception^40^. But how does adaptation to density affect the perception of numerosity? Does it imply that our brain perceives numerosity using sensory integration? We argue that the influence on numerosity perception by adaptation to density need not imply the presence of a sensory integration system for number perception. If our assumption that sensory cues such as texture and density are used to prepare the network to process numerosity is correct, it explains why adaptation to visual cues is also reflected in the tasks involving numerosity perception.

## Conclusion

To explore visual perception of numbers, we utilized a Recurrent Neural Network (RNN) with on-centre off-surround neural connections. The RNN takes input inspired by the Normalized Object Location Map (OLM)^18^ and produces output based on the mean activation of its network nodes. Our model successfully reproduces key findings from behavioral studies on Weber fraction, number comparison, and reaction time^37^. In this study, we expand the model by introducing two novel approaches. Firstly, we developed a technique to accurately estimate numbers by decoding the mean activation (Decoding Algorithm). Second, we implemented a mechanism to regulate network inhibition, enhancing the model’s performance across a wide range of numerosity (Selection Algorithm). In addition, we have introduced a saliency map based approach to normalize visual information, resulting in the generation of an Object Location Map (OLM) from a visual scene. To illustrate this concept, we utilized the Rare 2012^31^ bottom-up saliency model along with fundamental image processing techniques, including thresholding and normalization using OpenCV (Fig. 1).

Behavioral studies have provided evidence that our ability to perceive numerosity is influenced by the range of numbers involved. Within the subitizing range (1 to 4), we can rapidly and accurately enumerate or discriminate numbers. On the contrary, numbers in the estimation range (greater than 5) require more time, and their discrimination adheres to Weber’s law^5,6^. Recent research has revealed the presence of an additional ’elbow’ or inflection point at number 20, which introduces three distinct ranges of numbers with sudden changes in number cognition^21^. However, the underlying mechanisms behind these behavioral discontinuities remain poorly understood.

In our computational model, we utilize the mean steady-state activation of all neurons in the network as the network’s output. We assume that the mean activation should exhibit a monotonic relationship with the input numerosity to accurately encode the numerosity. However, to maintain this monotonic relationship, we find that multiple levels of inhibition strengths are required (see Fig. 3). By minimizing the total number of inhibition strengths needed to cover the extended range of numerosity effectively, we identify three specific inhibition strengths ($\beta =0.15, \beta =0.04, \beta =0.01$) that correspond to three distinct ranges of numbers (1:4, 5:17, and 21:50). Notably, these ranges closely align with those observed in behavioral studies^5,6,21^. This observation provides a new perspective on the potential mechanisms underlying the inflection points observed in numerosity perception. It suggests that the observed discontinuity in our perception of numbers does not necessarily require dedicated neural networks for each range of numbers.

In the latter part of our study, we proposed two potential methods to regulate network inhibition, allowing for more accurate estimations across a wider range of inputs. In the first method, we determined the level of inhibition that resulted in the network’s output being most sensitive to slight changes in the input. By selecting inhibition strengths based on this criterion, we achieved reasonable number estimates for both small and large numbers. This approach sets our network apart as one of the few computational models capable of operating effectively within an extended domain of numerosities (1 to 50) (see Fig. 5). In the second method, we proposed that an initial, albeit less precise, estimation of numerosity from the early visual pathways could assist in selecting an appropriate inhibition strength for the current input. This selected inhibition strength could then prepare the network to generate an accurate representation of numerosity based on a normalized input devoid of primary visual attributes. Thus, we advocate for a hybrid system that combines concepts from the Sensory Integration Theory (SIT) to obtain an initial guess of numerosity and the Approximate Number System (ANS) theory to refine the final numerosity estimate at deeper stages of the visual pathway. This section of our model is currently in a conceptual stage, and we are actively working on incorporating relevant SIT algorithms to computationally demonstrate this idea.

Our model provides a computational explanation for certain adaptation effects observed in psychophysics studies. We propose that adaptation to a specific numerosity range may arise due to imperfect and delayed regulation of network inhibition. The additional time required to find an appropriate network parameter could potentially contribute to a switch cost, leading to increased reaction times, as seen in Sengupta et. al.^37^. Furthermore, the decrease in accuracy immediately following a switch could be attributed to the use of a network parameter that is no longer suitable for the input. If our assumption holds that sensory cues such as texture and density are utilized to prepare the network to achieve more accurate estimates, it also offers an explanation for why adaptation to visual cues such as texture and size influences tasks related to numerosity^39^.

Our model of number perception has immense potential for further development. Because the model represents numerosity in a continuous manner, more research is needed to investigate the underlying processes that lead to the symbolic grounding of numbers. While our current findings have allowed us to make predictions regarding the roles of primary visual cues, it is crucial to develop a specific algorithm to computationally validate these predictions.

We recognize that biological neural networks are much more intricate than our simplified model, and we do not assert that the observed behavioral phenomena are exclusively explained by our network. However, our model offers valuable insights into the potential computations underlying various intriguing aspects of visual numerosity perception. These insights can contribute to the development of more biologically relevant models for visual number perception and number cognition as a whole.

## Data Availability

The datasets used and/or analyzed during the current study available from the corresponding author on reasonable request. This paper is based on a simulation based experiment where the data gets generated while simulating the network dynamics. If required, we would personally provide the code and required assistance to understand the generated data and hidden variables.
